# Finite element simulations of hybrid nano-Carreau Yasuda fluid with hall and ion slip forces over rotating heated porous cone

**DOI:** 10.1038/s41598-021-99116-z

**Published:** 2021-10-01

**Authors:** Umar Nazir, Muhammad Sohail, Mahmoud M. Selim, Hussam Alrabaiah, Poom Kumam

**Affiliations:** 1 Department of Applied Mathematics and Statistics, Institute of Space Technology, P.O. Box 2750, Islamabad, 44000 Pakistan; 2 Department of Mathematics, Al-Aflaj College of Science and Humanities Studies, Prince Sattam Bin Abdulaziz University, Al-Aflaj, 710-11912 Saudi Arabia; 3 Department of Mathematics, Suez Faculty of Science, Suez University, Suez, 34891 Egypt; 4 College of Engineering, Al Ain University, Al Ain, UAE; 5 Department of Mathematics, Tafila Technical University Tafila, At-Tafilah, Jordan; 6 Center of Excellence in Theoretical and Computational Science (TaCS-CoE) and KMUTT Fixed Point Research Laboratory, Room SCL 802 Fixed Point Laboratory, Science Laboratory Building, Departments of Mathematics, Faculty of Science, King Mongkut’s University of Technology Thonburi (KMUTT), 126 Pracha-Uthit Road, Bang Mod, Thung Khru, Bangkok, 10140 Thailand; 7 Department of Medical Research, China Medical University Hospital, China Medical University, Taichung, 40402 Taiwan

**Keywords:** Mathematics and computing, Nanoscience and technology

## Abstract

Involvement of hybrid nanoparticles a vital role to improve the efficiency of thermal systems. This report covers the utilization of different nanoparticles mixed in Carreau Yasuda material for the improvement of thermal performance. The configuration of flow situation is considered over a rotating porous cone by considering the Hall and Ion slip forces. Transport of momentum is considered to be in a rotating cone under generalized ohm’s law and heat transfer is presented by considering viscous dissipation, Joule heating and heat generation. Rheology of considered model is derived by engaging the theory proposed by Prandtl. Modeled complex PDEs are reduced into ODEs under similarity transformation. To study the physics behind this phenomenon, solution is essential. Here, FEM (Finite Element Method) is adopted to compute the solution. Furthermore, the grid independent study is reported with several graphs and tables which are prepared to note the influence of involved parameters on thermal and velocity fields. It is worth mentioning that heat transport is controlled via higher radiation parameter and it upsurges for Eckert number. Moreover, Hall and ion slip parameters are considered significant parameters to produce the enhancement in motion of fluid particles but speed of nano and hybrid nanoparticles becomes slow down versus large values of Forchheimer and Weissenberg numbers. Additionally, an enhancement in production of heat energy is addressed via large values of heat generation number and Eckert number while reduction in heat energy is occurred due to positive values of thermal radiation and Hall and ion slip parameters.

## Introduction

Flow over a rotating geometries got considerable attention by the researchers due to their wider applications in numerous technological developments instruments and appliances. Inclusion/mixing of hybrid nanoparticles is highly recommended by the engineers to improve the thermal performance. Many rheological relations have been proposed by the researchers to study transport phenomenon. An important relation of Carreau-Yasuda model^1–5^ is

$${\eta }_{CY}\left(\dot{\gamma }\right)={\mu }_{\infty }+\left({\mu }_{0}-{\mu }_{\infty }\right){\left[1+{\left(\Upsilon \dot{\gamma }\right)}^{d}\right]}^{\frac{n-1}{d}}.$$

For $\Upsilon =0$ or $n=1,$ Newtonian model is recovered. Due to diverse applications, this model got the remarkable attention and attraction by different researchers. For instance, Mahmood et al.^1^ presented the finite element based computational analysis on Carreau-Yasuda model in a cavity with obstacle. They plotted the behavior of influential fluid parameters and analyzed the tabular results for comparison purpose. They noted the depreciation in viscosity of Carreau-Yasuda material by improving the relaxation time. Steady and oscillatory flow behavior of Carreau-Yasuda material via Lattice Boltzmann procedure (LBP) was reported by Boyd and Buick^2^. They discovered the different behavior in flow of Carreau-Yasuda material under different situations and assumptions. Coclite et al.^3^ analyzed different impacts of Carreau-Yasuda material in a lid-driven cavity. Stability analysis for Carreau-Yasuda material obeying poiseulle flow phenomenon via Chebyshev polynomial tool (CPT) was explored by Pinarbasi and Liakopoulos^4^. Bio-convection phenomenon in radiated chemically reactive magnetized slip of Carreau-Yasuda material was examined by Waqas et al.^5^. They engaged BVP4C package (MATLAB COMPUTATIONAL PACKAGE) to compute solution of transformed modeled problem. Several important flow features have been captured against numerous influential parameters. Results have been compared as a limiting case current inspection with the published ones. They observed the decline in fluid velocity against Rayleigh number and it escalates for Weissenberg parameter. Also, augmentation in thermal field is recorded against slip parameter.

Thermal stability and mechanism of heat transportation is essential to study the thermal performance of nanoparticles. Researchers have presented several models for the thermpophysical features of nanoparticles and recorded their advantages and disadvantages. Inclusion of nanoparticles is a hot topic of research because of their vast applications. One cannot avoid the use of nanoparticles. These coated particles are used in different appliances and medical instruments for the treatment of patients suffering in different diseases. Several researchers paid attention on this direction. For instance, Darcy-Forchheimer flow of convective carbon water based nanofluid immersed in a stretching cylinder with viscous dissipation, radiation, variable thermal conductivity and obeying slip constraints was analyzed by Hayat et al.^6^ by engaging the model of thermophysical features proposed by Xue. Solution to the governing modeled expressions has been approximated by shooting procedure. They recorded the dual behavior of velocity and temperature fields against curvature parameter. Moreover, heat transportation rate escalates against radiation parameter, whereas depreciation in skin friction is noted for growing Eckert number. Electrically conducting radiative stretched flow of convective incompressible nanofluid with variable magnetic field was studied by Nayak et al.^7^ via shooting procedure. They presented the validation of obtained solution by comparing the results. They recorded the diminution in thermal field for higher convection parameter. Hady et al.^8^ presented the comparative analysis for convective nonlinear flow saturated in permeable surface via numerically procedure. They displayed several results against numerous influential parameters. They found the decrease in heat transportation rate for higher porosity parameter and an increase in skin friction. Nonlinear chemically reactive flow of Maxwell nanofluid past over a rotating stretched surface with activation energy was explored by Shafique et al.^9^. They used shooting method to obtain the solution of boundary layer transformed ODEs. They observed the influence of several emerging parameters through graphs and tabular data. They noted the enhancement in mass transfer rate for Schmidt number and depreciation in concentration field. Utilization of SWCNTs and MWCNTs to improve the thermal performance of engine oil and water based rotating viscous liquid with internal heating was examined by Rehman et al.^10^. They solved the resulting equations numerically and flow behavior is monitored through graphs and tabular data. They analyzed the higher skin friction and heat transfer rate for engine oil based mixture as compared with water based mixture for both MWCNTs and SWCNTs. Moreover, significant escalation in velocity field is recorded for higher volume fraction. Seth et al.^11^ worked on nonlinear mixed convective flow of viscous liquid past over a nonlinear stretched surface via FEM and OHAM. They considered velocity slip and performed regression analysis. They noticed the increase in velocity for stagnation parameter and decrease in thermal profile. Kandasamy et al.^12^ developed alumunia and copper based model to notice the thermal performance of mixed convective chemically reactive flow. They used the thermophysical model proposed by Magyari and Mamut. They noticed the several important features through plots. They monitored the rise in velocity for velocity slip parameter and opposite trend in concentration field. McCash et al.^13^ studied characteristics of viscous fluid inside two tubes using exact solution approach. Zidan et al.^14^ discussed the thermal aspects of blood flow in multiple stenosis. They used exact approach to know behavior of blood flow and entropy generation. Saleem et al.^15^ performed bio-mathematical scheme to know behavior of blood flow in artery (non-symmetric and symmetric stenosed) including Joule heating. McCash et al.^16^ modeled flow behavior of Peristaltic liquid inserting hybrid nanoparticles in an Elliptic Duct along with advancing boundaries. Rehman et al.^17^ highlighted thermal aspects in pseudoplastic liquid inserting nanoparticles over Riga heated surface considering thermophoresis diffusion and Brownian motion. They estimated surface force, flow and heat energy using various physical parameters and numerically solved by numerical scheme. Akhtar et al.^18^ discussed features of heat energy in non-Newtonian fluid including carbon nanotubes towards. They used exact solution scheme to know aspects of pressure gradient, heat energy and flow phenomena inside melting a vertical duct. Rizwana et al.^19^ scrutinized formulation of thermal aspects under the action of magnetic field over oscillating melting plate inserting nanoparticles along with convective boundary conditions. Yasin et al.^20^ discussed laminar flow in heated rods via finite element method approach. Ahmad et al.^21^ formulated micropolar liquid suspending hybrid nanoparticles using non-Fourier’s theory considering triple stratification. Yasin et al.^22^ used finite element approach to know aspects of Lorentz forces along with convective flow in adiabatic (enclosure). Hussain et al.^23^ formulated heat transfer in Carreau–yasuda liquid inserting nanoparticles towards melting surface. Nazir et al.^24^ simulated comparative results of hybrid nanoparticles in Williamson among nanoparticles and hybrid nanoparticles towards a meeting sheet using non-Fourier’s theory. In another survey, Nazir et al.^25^ discussed comparison analysis in Carreau liquid among variable and constant viscosity via non-Fourier’s theory numerically solved by FEA (finite element approach). Important studies contributing the modeling of several phenomena under different flow conditions are reported in^26–31^.

Available literature has no reported study by considering the inclusion of hybrid nanoparticles in Carreau-Yasuda model with dissipation effect and engagement of Hall and ion slip forces in rotating porous cone. This report will be used as a base for the researchers working further on Carreau-Yasuda model by engaging different physical effects. This draft is organized as: comprehensive literature survey is included in Sect. 1, modeling is mentioned in Sect. 2 along with important physical quantities, Sect. 3 contains the explanation of solution scheme, graphical and tabular results are reported in Sect. 4 and important results have been listed in Sect. 5.

In future endeavors this work will be extended by considering following important effects.

- Slip effects, variable viscosity (space dependent/shear rate dependent/concentration dependent/temperature dependent) and variable magnetic field;
- Mixed convection;
- Modified heat flux and radiation effect;
- Variable thermal conductivity;
- Space dependent heat source;
- Variable diffusion coefficient;
- Utilization of ternary hybrid nanoparticles mixture;
- Linear, nonlinear stretching sheets with and without porosity;
- CPU analysis of iteration of different schemes and comparative study.

**Nomenclature**

**Table:** 

Symbols/units	Used for	Symbols/units	Used for
$z,x,y$[m]	Space coordinates	$Ec$[no unit]	Eckert number
$u, v,w$[ms^−1^]	Velocity components	$Re$[no unit]	Reynolds number
$G$[Newton]	Gravitational force	$nf$	Nano-fluid
${U}_{w}$[ms^−1^]	Wall velocity	$We$[no unit]	Weissenberg number
$T$[kelvin]	Temperature field	${H}_{s}$	Heat generation number
${T}_{\infty }, {T}_{w}$[kelvin]	Ambient and wall temperatures	$Nu$	Nusselt number
${B}_{0}$[Oersted Ampere/meter]	Magnetic field strength	$Si{O}_{2}$	Silicon dioxide
${C}_{f}, {C}_{g}$	Skin friction coefficients	${T}_{0}$[kelvin]	Reference temperature
$k$[(W/(m⋅K))]	Thermal conductivity	Greek symbols	
$hnf,bf$	Hybrid nanofluid and base fluid	$\alpha$[radian]	Semi vehicle angle
${C}_{p}$[J kg^−1^ K^−1^]	Specific heat capacity	${\beta }_{i, }{\beta }_{e}$	Ion slip and Hall forces
$g,f$	Velocity components	$\phi , {\phi }_{2},{\phi }_{1}$	Volume fractions
${M}^{2}$	Magnetic field	$\theta$	Temperature
$Pr$[no unit]	Prandtl number	$\eta$	Independent variable
$l$[m]	Characteristic length	${\tau }_{xz}$	Wall shear stress
$n$	Power law index number	$\lambda$	Mixed convection parameter
$d$	Carreau Yasuda fluid number	$\Omega$	Radial velocity
${F}_{s}$	Inertia cofficient	$\nu$ m^2^ s^−1^	Kinematic viscosity
${F}_{r}$	Forchheimer number	$\rho$[kg m^−3^]	Fluid density
${N}_{r}$	Thermal radiation number	$\mu$[kg m^−1^ s^−1^]	Viscosity
${C}_{2}{H}_{6}{O}_{2}$	Ethylene glycol	$\sigma$[Sm^−1^]	Electrical conductivity
$Mo{S}_{2}$	Molybdenum dioxide	$\Gamma$	Time constant
PDEs	Partial differential equations	$\epsilon$	porosity number

## Formulation of heat transport model

**Figure 1:**
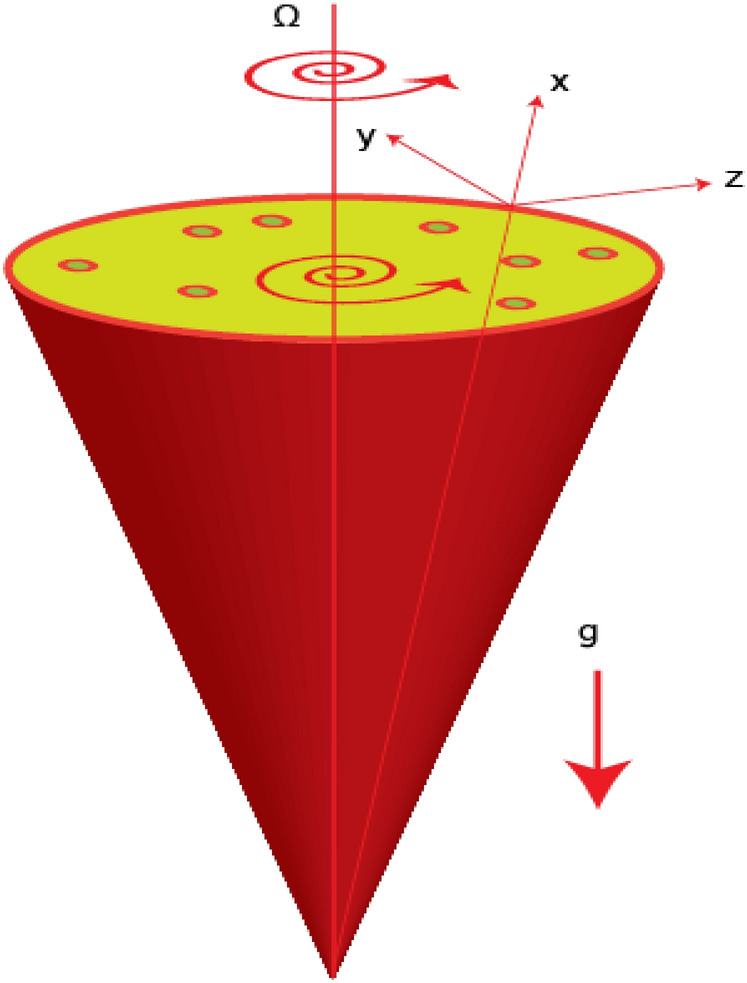
Flow behavior of hybrid nanoparticles.

**Figure 2:**
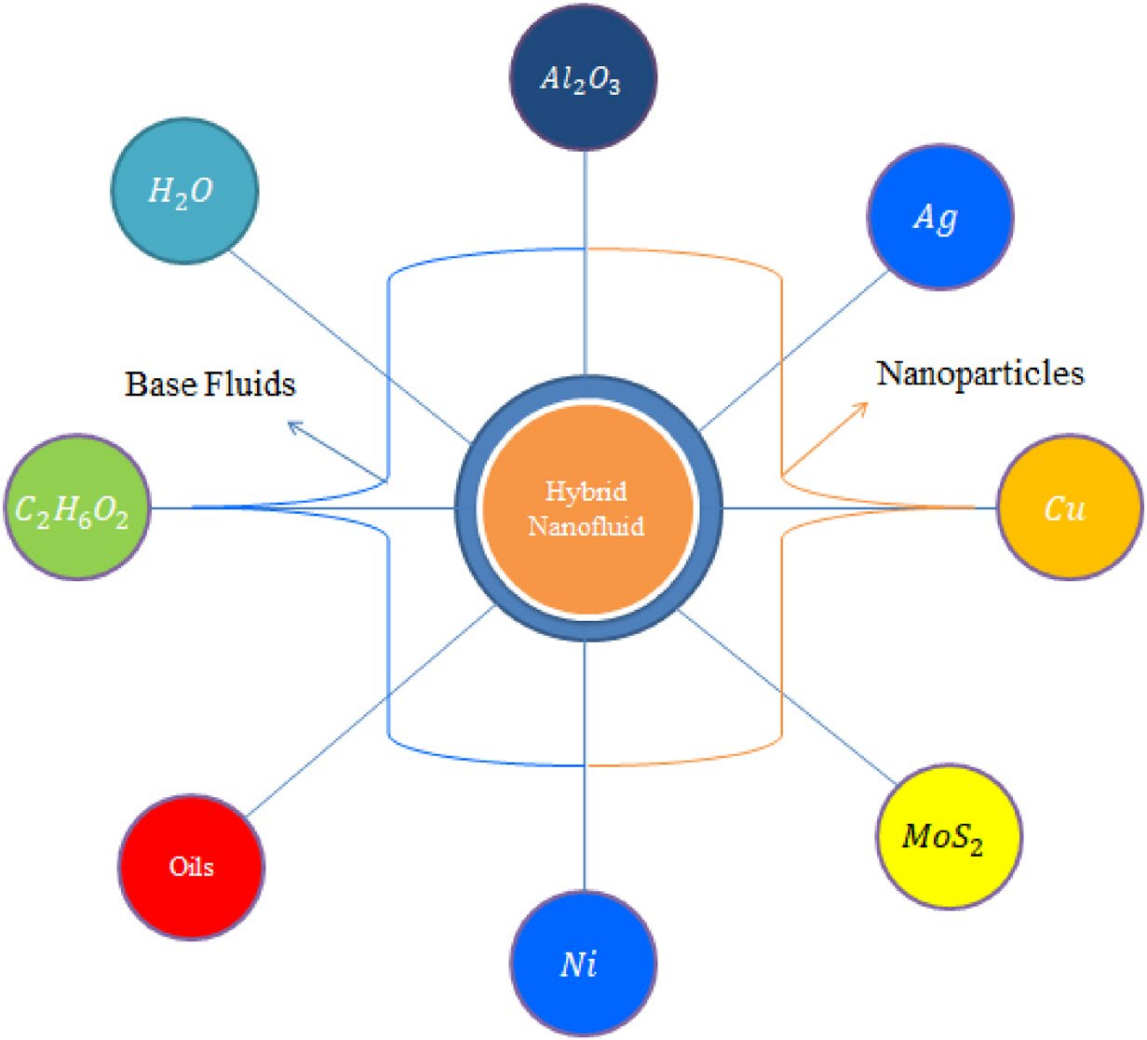
The sketching behavior of hybrid nanoparticles.

The simulations of transport of heat energy involving the dispersion of $Mo{S}_{2}$ and $Si{O}_{2}$ called hybrid nanofluid in Carreau Yasuda liquid past a porous rotating cone with variable wall temperate are performed. Physically, the rotation in flow of hybrid nanoparticles is occurred due to rotating of a cone while Hall and ion-slip currents are taken into account. The heated cone is designed as space coordinates $\left(x, y, z\right)$ are taken along $u, v$ and $w$ whereas x-axis is known as tangential direction, azimuthal and normal directions are called y- and z-axis. The bouncy forces are appeared due to gravitational force. Moreover, the impacts of Darcy's porous medium, Joule heating, viscous dissipation, thermal radiation and heat generation are modeled. The composition of $Mo{S}_{2}$ and $Si{O}_{2}$ is called hybrid nanoparticles while $Mo{S}_{2}$ is named as nanoparticles in base liquid (ethylene glycol). Physical flow transport phenomena are illustrated by Fig. 1. The sketching view of hybrid nanoparticles is considered by Fig. 2.  Thermal properties of hybrid nanoparticles is mentioned in table 1. The non-linear PDEs^32,33^ are developed using BLAs (boundary layer approximations) and present flow phenomena in mathematical mode is established as

1 $$\frac{{\partial \left( {xu} \right)}}{\partial x} + \frac{{\partial \left( {xv} \right)}}{\partial z} = 0,$$

2 $$\left. {\begin{array}{*{20}c} {u\frac{\partial u}{{\partial x}} + w\frac{\partial u}{{\partial z}} = \frac{{v^{2} }}{x} + \nu_{hnf} \left[ {\frac{{\partial^{2} u}}{{\partial z^{2} }} + {\Gamma }^{d} \left( {\frac{n - 1}{d}} \right)\left( {d + 1} \right)\frac{{\partial^{2} u}}{{\partial z^{2} }}\left( {\frac{\partial u}{{\partial z}}} \right)^{d} } \right] + G\beta \left( {T - T_{\infty } } \right)cos\alpha } \\ { + \frac{{B_{0}^{2} \sigma_{hnf} }}{{\rho_{hnf} \left[ {\left( {1 + \beta_{e} \beta_{i} } \right)^{2} + \beta_{e}^{2} } \right]}}\left[ {v\beta_{e} - \left( {1 + \beta_{e} \beta_{i} } \right)u} \right] - \frac{{\nu_{hnf} }}{{k^{*} }}F_{s} u - \frac{{F_{s} }}{{\left( {k^{*} } \right)^{1/2} }}u^{2} } \\ \end{array} } \right\},$$

3 $$\left. {\begin{array}{*{20}l} {u\frac{\partial v}{{\partial x}} + w\frac{\partial v}{{\partial z}} = \frac{uv}{x} + \nu_{hnf} \left[ {\frac{{\partial^{2} v}}{{\partial z^{2} }} + {\Gamma }^{d} \left( {\frac{n - 1}{d}} \right)\left( {d + 1} \right)\frac{{\partial^{2} v}}{{\partial z^{2} }}\left( {\frac{\partial v}{{\partial z}}} \right)^{d} } \right]} \hfill \\ { - \frac{{B_{0}^{2} \sigma_{hnf} }}{{\rho_{hnf} \left[ {\left( {1 + \beta_{e} \beta_{i} } \right)^{2} + \beta_{e}^{2} } \right]}}\left[ {u\beta_{e} + \left( {1 + \beta_{e} \beta_{i} } \right)v} \right] - \frac{{\nu_{hnf} }}{{k^{*} }}F_{s} v - \frac{{F_{s} }}{{\left( {k^{*} } \right)^{1/2} }}v^{2} } \hfill \\ \end{array} } \right\}$$

4 $$\left. {\begin{array}{*{20}l} {u\frac{\partial T}{{\partial x}} + w\frac{\partial T}{{\partial z}} = \frac{{k_{hnf} }}{{\left( {\rho c_{p} } \right)_{hnf} }}\left[ {\frac{{\partial^{2} T}}{{\partial z^{2} }} + \frac{{\sigma^{*} 16T_{\infty }^{3} }}{{3k^{*} }}\frac{{\partial^{2} T}}{{\partial z^{2} }}} \right] + \frac{{B_{0}^{2} \sigma_{hnf} }}{{\rho_{hnf} \left[ {\left( {1 + \beta_{e} \beta_{i} } \right)^{2} + \beta_{e}^{2} } \right]}}\left( {u^{2} + v^{2} } \right)} \hfill \\ { + \frac{{\mu_{hnf} }}{{\left( {\rho C_{p} } \right)_{hnf} }}\left[ {1 + \left\{ {\left( {\Gamma } \right)^{d} \left( {\frac{n - 1}{d}} \right)} \right\}\left( {\frac{\partial u}{{\partial z}}} \right)^{d} + \left( {\frac{\partial v}{{\partial z}}} \right)^{d} } \right]\left[ {\left( {\frac{\partial u}{{\partial z}}} \right)^{2} + \left( {\frac{\partial v}{{\partial z}}} \right)^{2} } \right] + Q_{0} \left( {T - T_{\infty } } \right)} \hfill \\ \end{array} } \right\}$$

**Table 1:** Thermal properties of hybrid nanoparticles with base fluid.

$Mo{S}_{2}/Si{O}_{2}$	${C}_{2}{H}_{6}{O}_{2}$	$Mo{S}_{2}$
${\rho }_{Mo{S}_{2}/Si{O}_{2}}=5060$	${\rho }_{{C}_{2}{H}_{6}{O}_{2}}=1113.5$	${\rho }_{Mo{S}_{2}}=2650$
${\left({C}_{p}\right)}_{Mo{S}_{2}/Si{O}_{2}}=397.746$	${\left({C}_{p}\right)}_{{C}_{2}{H}_{6}{O}_{2}}=2430$	${\left({C}_{p}\right)}_{Mo{S}_{2}}=730$
${k}_{Mo{S}_{2}/Si{O}_{2}}=34.5$	${k}_{{C}_{2}{H}_{6}{O}_{2}}=0.253$	${k}_{Mo{S}_{2}}=1.5$
${\sigma }_{Mo{S}_{2}/Si{O}_{2}}=1\times {10}^{-18}$	${\sigma }_{{C}_{2}{H}_{6}{O}_{2}}=4.3\times {10}^{-5}$	${\sigma }_{Mo{S}_{2}}=0.0005$

The BCs (boundary conditions)^32^ are simulated using concept of no-slip theory

5 $$\left. {\begin{array}{*{20}l} {u = 0, v = \Omega xsin\alpha , T = T_{w} , w = 0\; at\; z = 0} \hfill \\ {u \to 0, v \to 0,T \to T_{\infty }\; at\; z \to \infty } \hfill \\ \end{array} } \right\}.$$

The change of variables are constructed as

6 $$\left. {\begin{array}{*{20}c} {u = - \frac{{{\Omega }xsin\alpha }}{2}f^{\prime}, v = \Omega xsin\alpha g, w = \left( {{\Omega }\nu_{f} sin\alpha } \right)^{\frac{1}{2}} f} \\ {\theta = \frac{{T - T_{\infty } }}{{T_{w} - T_{\infty } }},\eta = z\sqrt {\frac{{{\Omega }sin\alpha }}{{\nu_{f} }}} ,T_{w} = T_{\infty } + \frac{{x\left( {T_{0} - T_{\infty } } \right)}}{l} } \\ \end{array} } \right\}.$$

The correlations of thermo-physical properties in nano and hybrid nanoparticles are

7 $$\left. {\begin{array}{*{20}l} {\begin{array}{*{20}l} {\rho _{{nf}} = \left( {1 - \phi } \right)\rho _{f} + \phi \rho _{s} ,\;\rho _{{hnf}} = \left[ {\left( {1 - \phi _{2} } \right)\left\{ {\left( {1 - \phi _{1} } \right)\rho _{f} + \phi _{1} \rho _{{s_{1} }} } \right\}} \right] + \phi _{2} \rho _{{s_{2} }} ~} \hfill \\ {\left( {\rho C_{p} } \right)_{{nf}} = \left( {1 - \phi } \right)\left( {\rho C_{p} } \right)_{f} + \phi \left( {\rho C_{p} } \right)_{s} ,~\left( {\rho C_{p} } \right)_{{hnf}} = \left[ {\left( {1 - \phi _{2} } \right)\left\{ {\left( {1 - \phi _{1} } \right)\left( {\rho C_{p} } \right)_{f} + \phi _{1} \left( {\rho C_{p} } \right)_{{s_{1} }} } \right\}} \right]~} \hfill \\ \end{array} } \hfill \\ { + \phi _{1} \left( {\rho C_{p} } \right)_{{s_{2} }} } \hfill \\ \end{array} } \right\},$$

8 $$\left. {\begin{array}{*{20}l} {\mu _{{nf}} = \frac{{\mu _{f} }}{{\left( {1 - \phi } \right)^{{2.5}} }},\;\mu _{{nf}} = \frac{{\mu _{f} }}{{\left( {1 - \phi _{2} } \right)^{{2.5}} \left( {1 - \phi _{1} } \right)^{{2.5}} }},~\;\frac{{k_{{nf}} }}{{k_{f} }} = \left\{ {\frac{{k_{s} + \left( {n + 1} \right)k_{f} - \left( {n - 1} \right)\phi \left( {k_{f} - k_{s} } \right)}}{{k_{s} + \left( {n - 1} \right)k_{f} + \phi \left( {k_{f} - k_{s} } \right)}}} \right\}} \hfill \\ {\frac{{k_{{hnf}} }}{{k_{{bf}} }} = \left\{ {\frac{{k_{{s_{2} }} + \left( {n - 1} \right)k_{{bf}} - \left( {n - 1} \right)\phi _{2} \left( {k_{{bf}} - k_{{s_{2} }} } \right)}}{{k_{{s_{2} }} + \left( {n - 1} \right)k_{{bf}} - \phi _{2} \left( {k_{{bf}} - k_{{s_{2} }} } \right)}}} \right\},\;\frac{{\sigma _{{hnf}} }}{{\sigma _{f} }} = \left( {1 + \frac{{3\left( {\sigma - 1} \right)\phi }}{{\left( {\sigma + 2} \right) - \left( {\sigma - 1} \right)\phi }}} \right)} \hfill \\ {\frac{{\sigma _{{hnf}} }}{{\sigma _{f} }} = \left( {\frac{{\sigma _{{s_{2} }} + 2\sigma _{f} - 2\phi _{2} \left( {\sigma _{{bf}} - \sigma _{{s_{2} }} } \right)}}{{\sigma _{{s_{2} }} + 2\sigma _{f} + \phi _{2} \left( {\sigma _{{bf}} - \sigma _{{s_{2} }} } \right)}}} \right),\frac{{\sigma _{{bf}} }}{{\sigma _{f} }} = \left( {\frac{{\sigma _{{s_{1} }} + 2\sigma _{f} - 2\phi _{1} \left( {\sigma _{f} - \sigma _{{s_{1} }} } \right)}}{{\sigma _{{s_{1} }} + 2\sigma _{f} + \phi _{1} \left( {\sigma _{f} - \sigma _{{s_{1} }} } \right)}}} \right)} \hfill \\ \end{array} } \right\}$$

Equations (1–5) are transformed into dimensionless Eqs. (7–9) using Eq. (6)

9 $$\left. {\begin{array}{*{20}l} {f^{{\prime \prime \prime }} + \frac{{\nu _{f} }}{{\nu _{{hnf}} }}\left( {\frac{1}{2}\left( {f^{\prime } } \right)^{2} - ff^{{\prime \prime }} - 2g^{2} - 2\lambda \theta } \right) - \frac{{M^{2} \left( {1 - \phi _{1} } \right)^{{2.5}} \left( {1 - \phi _{2} } \right)^{{2.5}} }}{{\left( {1 + \beta _{e} \beta _{i} } \right)^{2} + \beta _{e}^{2} }}\left[ {2\beta _{e} g + \left( {1 + \beta _{e} \beta _{i} } \right)f^{\prime } } \right]} \hfill \\ { + \left( {We} \right)^{d} \frac{{\left( {n - 1} \right)\left( {d + 1} \right)}}{d}f^{{\prime \prime \prime }} \left( {ff^{{\prime \prime }} } \right)^{d} + \epsilon f^{\prime } - H_{1} F_{r} \left( {f^{\prime } } \right)^{2} = 0,} \hfill \\ {f\left( 0 \right) = f^{\prime } \left( 0 \right) = 0,\;f^{\prime } \left( \infty \right) = 0,} \hfill \\ \end{array} } \right\}$$

10 $$\left. {\begin{array}{*{20}l} {g^{{\prime \prime }} + \frac{{\nu _{f} }}{{\nu _{{hnf}} }}\left( {gf^{\prime } - fg^{\prime } } \right) - \frac{{M^{2} \left( {1 - \phi _{1} } \right)^{{2.5}} \left( {1 - \phi _{2} } \right)^{{2.5}} }}{{\left( {1 + \beta _{e} \beta _{i} } \right)^{2} + \beta _{e}^{2} }}\left[ { - \frac{1}{2}\beta _{e} f^{\prime } + \left( {1 + \beta _{e} \beta _{i} } \right)g} \right]} \hfill \\ { + \left( {We} \right)^{d} \frac{{\left( {n - 1} \right)\left( {d + 1} \right)}}{d}g^{{\prime \prime }} \left( {g^{\prime } } \right)^{d} - \epsilon g - H_{1} F_{r} \left( g \right)^{2} = 0,} \hfill \\ { { + g(0) = 1,\theta (\infty ) = 0,} } \hfill \\ \end{array} } \right\}$$

11 $$\left. {\begin{array}{*{20}c} {\left( {1 + \frac{4}{{3N_{r} }}} \right)\theta ^{{''}} + \frac{{k_{f} }}{{k_{{hnf}} }}\frac{{\left( {\rho c_{p} } \right)_{{hnf}} }}{{\left( {\rho c_{p} } \right)_{f} }}Pr\left( {\frac{1}{2}f^{\prime } \theta - f\theta ^{\prime } } \right) + \frac{{k_{f} }}{{k_{{hnf}} }}\frac{{\left( {1 - \phi _{1} } \right)^{{ - 2.5}} PrEcM^{2} }}{{\left( {1 - \phi _{2} } \right)^{{2.5}} \left[ {\left( {1 + \beta _{e} \beta _{i} } \right)^{2} + \beta _{e}^{2} } \right]}}\left[ {\frac{1}{4}\left( {f^{\prime } } \right)^{2} + \left( g \right)^{2} } \right]} \\ {\frac{{k_{f} }}{{k_{{hnf}} }}\frac{{PrEc}}{{\left( {1 - \phi _{1} } \right)^{{2.5}} \left( {1 - \phi _{2} } \right)^{{2.5}} }}\left[ {1 + \frac{{n - 1}}{d}\left( {We} \right)^{d} \left( {\frac{1}{4}\left( {f^{{\prime \prime }} } \right)^{d} + \left( {g^{\prime } } \right)^{d} } \right)} \right]\left( {\frac{1}{4}\left( {f^{{\prime \prime }} } \right)^{2} + \left( {g^{\prime } } \right)^{2} } \right) + \frac{{k_{f} }}{{k_{{hnf}} }}H_{s} Pr\theta = 0,} \\ {\theta \left( 0 \right) = 1,~\theta \left( \infty \right) = 0} \\ \end{array} } \right\}$$

### Physical quantities

The dimensionless parameters of present problem are defined as

$$\begin{aligned} Ec & = \frac{{xl\left( {{\Omega }sin\alpha } \right)^{2} }}{{\left( {C_{p} } \right)_{f} \left( {T_{0} - T_{w} } \right)}},\;\lambda = \frac{{g\left( {T_{0} - T_{w} } \right)l\beta cos\alpha }}{{{\Omega }sin\alpha \left( {\nu_{f} } \right)^{2} }}, \;M^{2} = \frac{{B_{0}^{2} \sigma_{hnf} }}{{\rho_{f} {\Omega }sin\alpha }}, \\ Pr & = \frac{{\mu_{f} \left( {C_{p} } \right)_{f} }}{{k_{f} }},\;H_{s} = \frac{{Q_{0} }}{{sin\alpha {\Omega }\left( {\rho C_{p} } \right)_{f} }},\;F_{r} = \frac{{F_{s} x}}{{\left( {k^{*} } \right)^{1/2} }}, \;\epsilon = \frac{{\nu_{f} F_{s} }}{{{\Omega }sin\alpha }},\;N_{r} = \frac{{k^{*} k_{f} }}{{4\sigma^{*} \left( {T_{\infty } } \right)^{3} }}. \\ \end{aligned}$$

Shear stresses in view of y- and x-directions are expressed as

$$\begin{aligned} C_{f} & = \frac{{2\tau_{xz} |_{z = 0} }}{{\rho_{f} \left( {{\Omega }xsin\alpha } \right)^{2} }}, C_{g} = \frac{{2\tau_{yz} |_{z = 0} }}{{\rho_{f} \left( {{\Omega }xsin\alpha } \right)^{2} }}, \\ \left( {Re} \right)^{1/2} C_{f} & = \frac{ - 1}{{\left( {1 - \phi_{1} } \right)^{2.5} \left( {1 - \phi_{2} } \right)^{2.5} }}\left[ {1 + \frac{n - 1}{d}\left( {Wef^{\prime\prime}\left( 0 \right)} \right)^{d} } \right]f^{\prime\prime}\left( 0 \right), \\ \left( {Re} \right)^{1/2} C_{g} & = \frac{ - 1}{{\left( {1 - \phi_{1} } \right)^{2.5} \left( {1 - \phi_{2} } \right)^{2.5} }}\left[ {1 + \frac{n - 1}{d}\left( {Weg^{\prime}\left( 0 \right)} \right)^{d} } \right]g^{\prime}\left( 0 \right). \\ \end{aligned}$$

The Nusselt number is constructed as

$$Nu = \frac{{xQ_{w} }}{{k_{f} \left( {T - T_{\infty } } \right)}}, Q_{w} = - k_{hnf} \frac{\partial T}{{\partial z}},$$

$$\left( {Re} \right)^{ - 1/2} Nu = \frac{{ - k_{hnf} }}{{k_{f} }}\theta^{\prime}\left( 0 \right).$$

The local Reynolds number is $Re\left(=\frac{{x}^{2}\Omega sin\alpha }{{\nu }_{f}}\right)$.

## Numerical method for solution

Weighted residual Galerkin approach (WRGA) is implemented to simulate numerical values of Eqs. (9–11). Here, ${f}^{^{\prime}}=F$ is considered to formulate the required residuals. The following description is discussed below.

### Division of problem domain

The domain of the problem is broken into 300 elements whereas weak forms are developed using the weighted residual integrals. Linear polynomial is made over each 300 elements of domain. Weights functions are multiplied along with residuals and integration is taken. The approximation computations of $f, \theta$ and $F$ are defined below. So the weighted residuals described in^24,25,30,31,34^ are

$${\int }_{{\eta }_{e}}^{{\eta }_{e+1}}We\left[{f}^{^{\prime}}-F\right]d\eta =0,$$

$${\int }_{{\eta }_{e}}^{{\eta }_{e+1}}{w}_{1}\left[\begin{array}{c}{F}^{{{\prime\prime}}}+\frac{{\nu }_{f}}{{\nu }_{hnf}}\left(\frac{1}{2}{\left(F\right)}^{2}-f{F}^{^{\prime}}-2{g}^{2}-2\lambda \theta \right)\\ -\frac{{M}^{2}{\left(1-{\phi }_{1}\right)}^{2.5}{\left(1-{\phi }_{2}\right)}^{2.5}}{{\left(1+{\beta }_{e}{\beta }_{i}\right)}^{2}+{\beta }_{e}^{2}}\left[2{\beta }_{e}g+\left(1+{\beta }_{e}{\beta }_{i}\right)F\right]\\ +{\left(We\right)}^{d}\frac{\left(n-1\right)\left(d+1\right)}{d}{F}^{{{\prime\prime}}}{\left(F{^{\prime}}\right)}^{d}+\epsilon F-{H}_{1}{F}_{r}{\left(F\right)}^{2}\end{array}\right]d\eta =0,$$

$${\int }_{{\eta }_{e}}^{{\eta }_{e+1}}{w}_{2}\left[\begin{array}{c}{g}^{{{\prime\prime}}}+\frac{{\nu }_{f}}{{\nu }_{hnf}}\left(g{f}^{^{\prime}}-fg{^{\prime}}\right)+{\left(We\right)}^{d}\frac{\left(n-1\right)\left(d+1\right)}{d}{g}^{{{\prime\prime}}}{\left({g}^{^{\prime}}\right)}^{d}-\epsilon g\\ -\frac{{M}^{2}{\left(1-{\phi }_{1}\right)}^{2.5}{\left(1-{\phi }_{2}\right)}^{2.5}}{{\left(1+{\beta }_{e}{\beta }_{i}\right)}^{2}+{\beta }_{e}^{2}}\left[-\frac{1}{2}{\beta }_{e}f{^{\prime}}+\left(1+{\beta }_{e}{\beta }_{i}\right)g\right]-{H}_{1}{F}_{r}{\left({f}^{^{\prime}}\right)}^{2}\end{array}\right]d\eta =0,$$

$${\int }_{{\eta }_{e}}^{{\eta }_{e+1}}{w}_{3}\left[\begin{array}{c}{g}^{{{\prime\prime}}}+\frac{{\nu }_{f}}{{\nu }_{hnf}}\left(g{f}^{^{\prime}}-f{^{\prime}}g{^{\prime}}\right)+{\left(We\right)}^{d}\frac{\left(n-1\right)\left(d+1\right)}{d}{g}^{{{\prime\prime}}}{\left({g}^{^{\prime}}\right)}^{d}-\epsilon g\\ -\frac{{M}^{2}{\left(1-{\phi }_{1}\right)}^{2.5}{\left(1-{\phi }_{2}\right)}^{2.5}}{{\left(1+{\beta }_{e}{\beta }_{i}\right)}^{2}+{\beta }_{e}^{2}}\left[-\frac{1}{2}{\beta }_{e}F+\left(1+{\beta }_{e}{\beta }_{i}\right)g\right]-{H}_{1}{F}_{r}{\left(F\right)}^{2}\end{array}\right]d\eta =0,$$

$${\int }_{{\eta }_{e}}^{{\eta }_{e+1}}{w}_{4}\left[\begin{array}{c}{\left(1+\epsilon \theta \right)\theta }^{{{\prime\prime}}}+\frac{{k}_{f}}{{k}_{hnf}}\frac{{\left(\rho {c}_{p}\right)}_{hnf}}{{\left(\rho {c}_{p}\right)}_{f}}Pr\left(\frac{1}{2}F\theta -f{\theta }^{^{\prime}}\right)+\frac{{k}_{f}}{{k}_{hnf}}{H}_{s}Pr\theta \\ +\frac{{k}_{f}}{{k}_{hnf}}\frac{PrEc{M}^{2}}{{\left(1+{\beta }_{e}{\beta }_{i}\right)}^{2}+{\beta }_{e}^{2}}\left(\frac{1}{4}{\left(F\right)}^{2}+{\left({g}^{^{\prime}}\right)}^{2}\right)\\ \frac{{k}_{f}}{{k}_{hnf}}\frac{PrEc}{{\left(1-{\phi }_{1}\right)}^{2.5}{\left(1-{\phi }_{2}\right)}^{2.5}}\left(\frac{1}{4}{\left(F{^{\prime}}\right)}^{2}+{\left({g}{{^{\prime\prime}}}\right)}^{2}\right)\end{array}\right]d\eta =0,$$

Here, ${w}_{1}, {w}_{2}, {w}_{3}$ and ${w}_{4}$ are weight functions. The unknown variables $f, F,g$ and $\theta$ are considered as

$$f=\sum_{j=1}^{2}{f}_{i}{\psi }_{j}, F=\sum_{j=1}^{2}{F}_{i}{\psi }_{j},\theta =\sum_{j=1}^{2}{\theta }_{i}{\psi }_{j}, g=\sum_{j=1}^{2}{g}_{i}{\psi }_{j},$$

### Assembly development

Assembly procedure plays a vital role for development of boundary vector, source vector and stiffness matrix. Further, it is used to generate the global stiffness matrix while Picard linearization approach makes linearization in non-linear equations. Hence, local stiffness elements are

$${K}_{ij}^{11}={\int }_{{\eta }_{e}}^{{\eta }_{e+1}}{\uppsi }_{i}\left(\frac{d{\uppsi }_{j}}{d\eta }\right)d\eta , {K}_{ij}^{12}=-{\int }_{{\eta }_{e}}^{{\eta }_{e+1}}{\uppsi }_{i}\left({\uppsi }_{j}\right)d\eta ,{K}_{ij}^{13}=0, {K}_{ij}^{14}=0, {b}_{i}^{1}=0,$$

$${K}_{ij}^{21}=0,{K}_{ij}^{23}={\int }_{{\eta }_{e}}^{{\eta }_{e+1}}\left[{\uppsi }_{j}{\uppsi }_{i}2\overline{g }+\frac{{M}^{2}{\left(1-{\phi }_{1}\right)}^{2.5}{\left(1-{\phi }_{2}\right)}^{2.5}}{{\left(1+{\beta }_{e}{\beta }_{i}\right)}^{2}+{\beta }_{e}^{2}}2{\beta }_{e}{\uppsi }_{i}\left({\uppsi }_{j}\right)\right]d\eta ,$$

$${K}_{ij}^{22}={\int }_{{\eta }_{e}}^{{\eta }_{e+1}}\left[\begin{array}{c}-\left(1++{\left(We\right)}^{d}\frac{\left(n-1\right)\left(d+1\right)}{d}{\left(\overline{{F }^{^{\prime}}}\right)}^{d}\right)\frac{d{\uppsi }_{i}}{d\eta }\frac{d{\uppsi }_{j}}{d\eta }\\ -\frac{{M}^{2}{\left(1-{\phi }_{1}\right)}^{2.5}{\left(1-{\phi }_{2}\right)}^{2.5}}{{\left(1+{\beta }_{e}{\beta }_{i}\right)}^{2}+{\beta }_{e}^{2}}\left[\left(1+{\beta }_{e}{\beta }_{i}\right){\uppsi }_{j}{\uppsi }_{i}\right]\\ +\epsilon {\uppsi }_{j}{\uppsi }_{i}-{H}_{1}{F}_{r}\overline{H}{\uppsi }_{j}{\uppsi }_{i}+\frac{{\nu }_{f}}{{\nu }_{hnf}}\left(\frac{1}{2}\overline{H}{\uppsi }_{j}{\uppsi }_{i}-\overline{f}{\uppsi }_{i}\frac{d{\uppsi }_{j}}{d\eta }\right)\end{array}\right]d\eta ,$$

$${K}_{ij}^{24}={\int }_{{\eta }_{e}}^{{\eta }_{e+1}}\left[-\frac{{\nu }_{f}}{{\nu }_{hnf}}\left(2\lambda {\uppsi }_{j}{\uppsi }_{i}\right)\right]d\eta , {b}_{i}^{2}=0,{K}_{ij}^{32}=0,{K}_{ij}^{34}=0,$$

$${K}_{ij}^{33}=-{\int }_{{\eta }_{e}}^{{\eta }_{e+1}}\left[\begin{array}{c}-\left(1+{\left(We\right)}^{d}\frac{\left(n-1\right)\left(d+1\right)}{d}{\left({\uppsi }_{i}\frac{d{\uppsi }_{j}}{d\eta }\right)}^{d}\right)\frac{d{\uppsi }_{i}}{d\eta }\frac{d{\uppsi }_{j}}{d\eta }\\ -\frac{{M}^{2}{\left(1-{\phi }_{1}\right)}^{2.5}{\left(1-{\phi }_{2}\right)}^{2.5}}{{\left(1+{\beta }_{e}{\beta }_{i}\right)}^{2}+{\beta }_{e}^{2}}\left[\left(1+{\beta }_{e}{\beta }_{i}\right)\right]{\uppsi }_{j}{\uppsi }_{i}-\epsilon {\uppsi }_{j}{\uppsi }_{i}\\ {-H}_{1}{F}_{r}\overline{g}{\uppsi }_{j}{\uppsi }_{i}\end{array}\right]d\eta , {b}_{i}^{3}=0,$$

$${K}_{ij}^{31}=-{\int }_{{\eta }_{e}}^{{\eta }_{e+1}}\left[\frac{{M}^{2}{\left(1-{\phi }_{1}\right)}^{2.5}{\left(1-{\phi }_{2}\right)}^{2.5}}{{\left(1+{\beta }_{e}{\beta }_{i}\right)}^{2}+{\beta }_{e}^{2}}\frac{1}{2}{\beta }_{e}{\uppsi }_{j}{\uppsi }_{i}\right]d\eta , {b}_{i}^{4}=0,$$

$${K}_{ij}^{44}=-{\int }_{{\eta }_{e}}^{{\eta }_{e+1}}\left[\begin{array}{l}-\left(1+\epsilon \theta \right)\frac{d{\uppsi }_{i}}{d\eta }\frac{d{\uppsi }_{j}}{d\eta }+\frac{{k}_{f}}{{k}_{hnf}}\frac{{\left(\rho {c}_{p}\right)}_{hnf}}{{\left(\rho {c}_{p}\right)}_{f}}Pr\left(\frac{1}{2}\overline{F}{\uppsi }_{j}{\uppsi }_{i}-\overline{f}{\uppsi }_{i}\frac{d{\uppsi }_{j}}{d\eta }\right)\\ +\frac{{k}_{f}}{{k}_{hnf}}{H}_{s}Pr{\uppsi }_{j}{\uppsi }_{i}\end{array}\right]d\eta ,$$

$${K}_{ij}^{41}=-{\int }_{{\eta }_{e}}^{{\eta }_{e+1}}\left[\frac{{k}_{f}}{{k}_{hnf}}\frac{PrEc{M}^{2}}{{\left(1+{\beta }_{e}{\beta }_{i}\right)}^{2}+{\beta }_{e}^{2}}\frac{1}{4}\overline{F}{\uppsi }_{j}{\uppsi }_{i}+\frac{{k}_{f}}{{k}_{hnf}}\frac{PrEc}{{\left(1-{\phi }_{1}\right)}^{2.5}{\left(1-{\phi }_{2}\right)}^{2.5}}\stackrel{-}{F{^{\prime}}}{\uppsi }_{i}\frac{d{\uppsi }_{j}}{d\eta }\right]d\eta ,$$

$${K}_{ij}^{43}=-{\int }_{{\eta }_{e}}^{{\eta }_{e+1}}\left[\begin{array}{c}\frac{{k}_{f}}{{k}_{hnf}}\frac{PrEc{M}^{2}}{{\left(1+{\beta }_{e}{\beta }_{i}\right)}^{2}+{\beta }_{e}^{2}}\frac{1}{4}\overline{{g }^{^{\prime}}}{\uppsi }_{j}\frac{d{\uppsi }_{j}}{d\eta }\\ -\frac{{k}_{f}}{{k}_{hnf}}\frac{PrEc}{{\left(1-{\phi }_{1}\right)}^{2.5}{\left(1-{\phi }_{2}\right)}^{2.5}}\frac{d{\uppsi }_{i}}{d\eta }\frac{d{\uppsi }_{j}}{d\eta }\end{array}\right]d\eta ,{K}_{ij}^{41}=0,$$

System of non-linear (algebraic equations) is modeled with help of assembly procedure.

$$Mt\left(f, {f}^{^{\prime}}, \theta \right)\left(\begin{array}{c}f\\ \theta \end{array}\right)=\left[\dot{F}\right],$$

$\dot{F}$(force vector), $Mt$ (global stiffness matrix) and force vector ($\dot{F}$) and unknown nodal values $$\left(\begin{array}{c}f\\ \theta \end{array}\right).$$

### Convergence analysis

The error is established as

$${E}_{er}=|{\chi }^{i}-{\chi }^{i-1}|$$

and range of convergence is noticed as

$$Max\left|{\chi }^{i}-{\chi }^{i-1}\right|<{10}^{-8}.$$

It is mentioned that system of linear equations is simulated iteratively according computational tolerance (${10}^{-8}$).

### Grid independent investigation

FEM code is designed in Maple 18 while [0, 8] is called computational domain. Table 2 is performed as grid independent analysis for 300 elements and solution becomes converge at mid of each 300 elements.

**Table 2:** Mesh-free simulations of velocities and temperature via 300 elements^34^.

Number of elements	$f{^{\prime}}\left(\frac{{\eta }_{\infty }}{2}\right)$	$g\left(\frac{{\eta }_{\infty }}{2}\right)$	$\theta \left(\frac{{\eta }_{\infty }}{2}\right)$
30	0.2397335394	0.05453547774	0.1933024653
60	0.2232020794	0.05200805054	0.1819254898
90	0.2177959068	0.05117858942	0.1782491777
120	0.2151131380	0.05076561105	0.1764321953
150	0.2135099778	0.05051829239	0.1753486293
180	0.2124439114	0.05035356891	0.1746289887
210	0.1875839826	0.04551373254	0.1595173162
240	0.2111142682	0.05014775377	0.1737326708
270	0.2106720780	0.05007924754	0.1734348231
300	0.2103180036	0.05002488541	0.1731961042

### Validation of results

It is noticed that results of present problem is verified with published study by Malik et al.^32^ considering ${H}_{s}=0, M=0.002, {F}_{r}=0, \epsilon =0, Ec=0, Pr=0.7, {\beta }_{e}=0, {\beta }_{i}=0, {\phi }_{1}=0, {\phi }_{2}=0.$ (Table 3)

**Table 3:** Validation of present results for skin friction coefficients and temperature gradient.

$\lambda$	Malik et al.^32^			present numerical values		
	${\left(Re\right)}^{1/2}{C}_{f}$	${\left(Re\right)}^{1/2}{C}_{g}$	${\left(Re\right)}^{-1/2}Nu$	${\left(Re\right)}^{1/2}{C}_{f}$	${\left(Re\right)}^{1/2}{C}_{g}$	${\left(Re\right)}^{-1/2}Nu$
0.0	1.0253	0.6153	0.4295	1.0248	0.6149	0.4291
1	2.2007	0.8492	0.6121	2.2003	0.8381	0.6130
10	8.5041	1.3990	1.0097	8.5039	1.3973	1.0088

## Results and discussion

In this section, the characterizations of heat transport phenomena in Carreau Yasuda liquid carrying nanoparticles and hybrid nanoparticles over a porous heated cone. The transport of heat energy takes place in terms of thermal radiation and viscous dissipation under the action of ion slip and Hall forces. The strong technique is used to capture the results in terms of tables and graphs. The detail study of current model is addressed as:

### Graphical simulations of fluid motion

**Figure 3:**
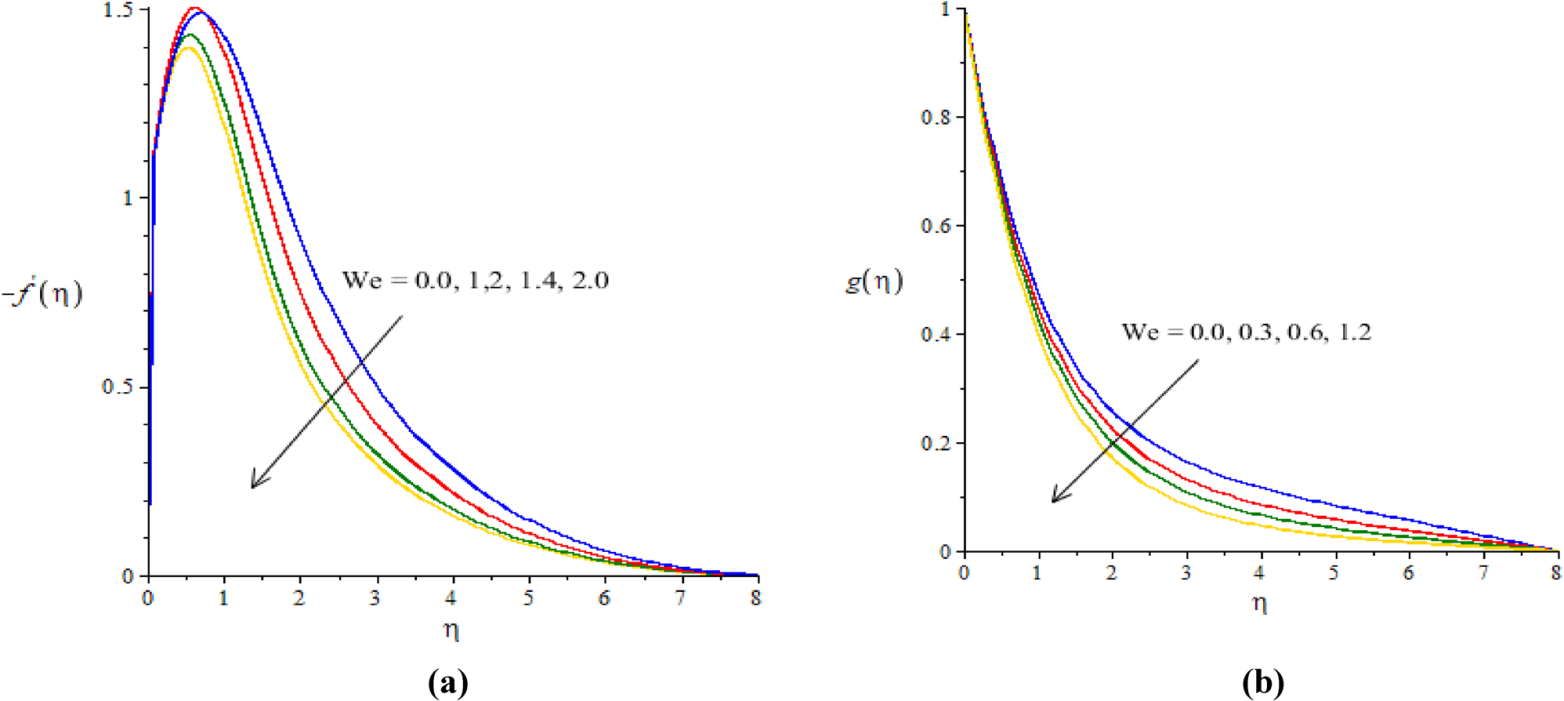
**(a, b)** The graphical view of velocities versus $We.$

**Figure 4:**
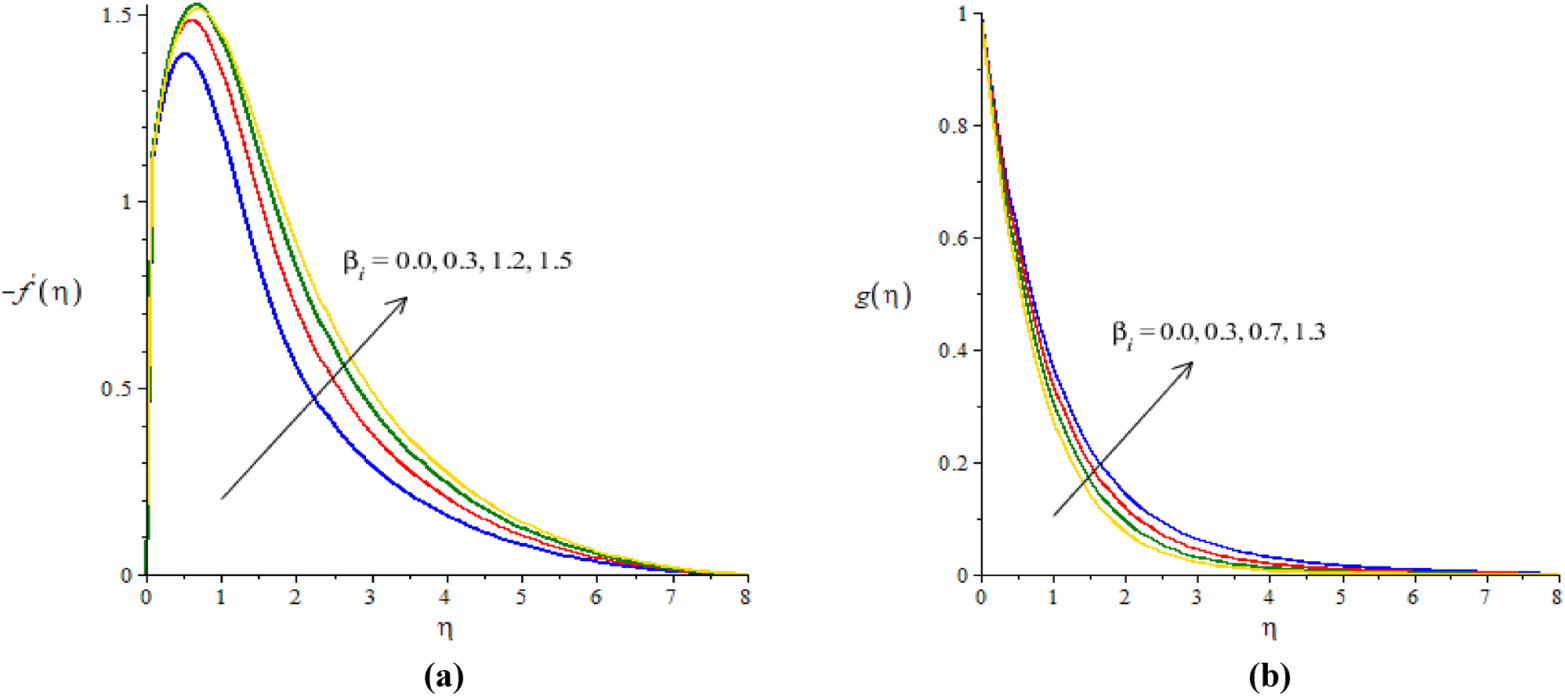
**(a,b)** The graphical view of velocities versus ${\beta }_{i}.$

**Figure 5:**
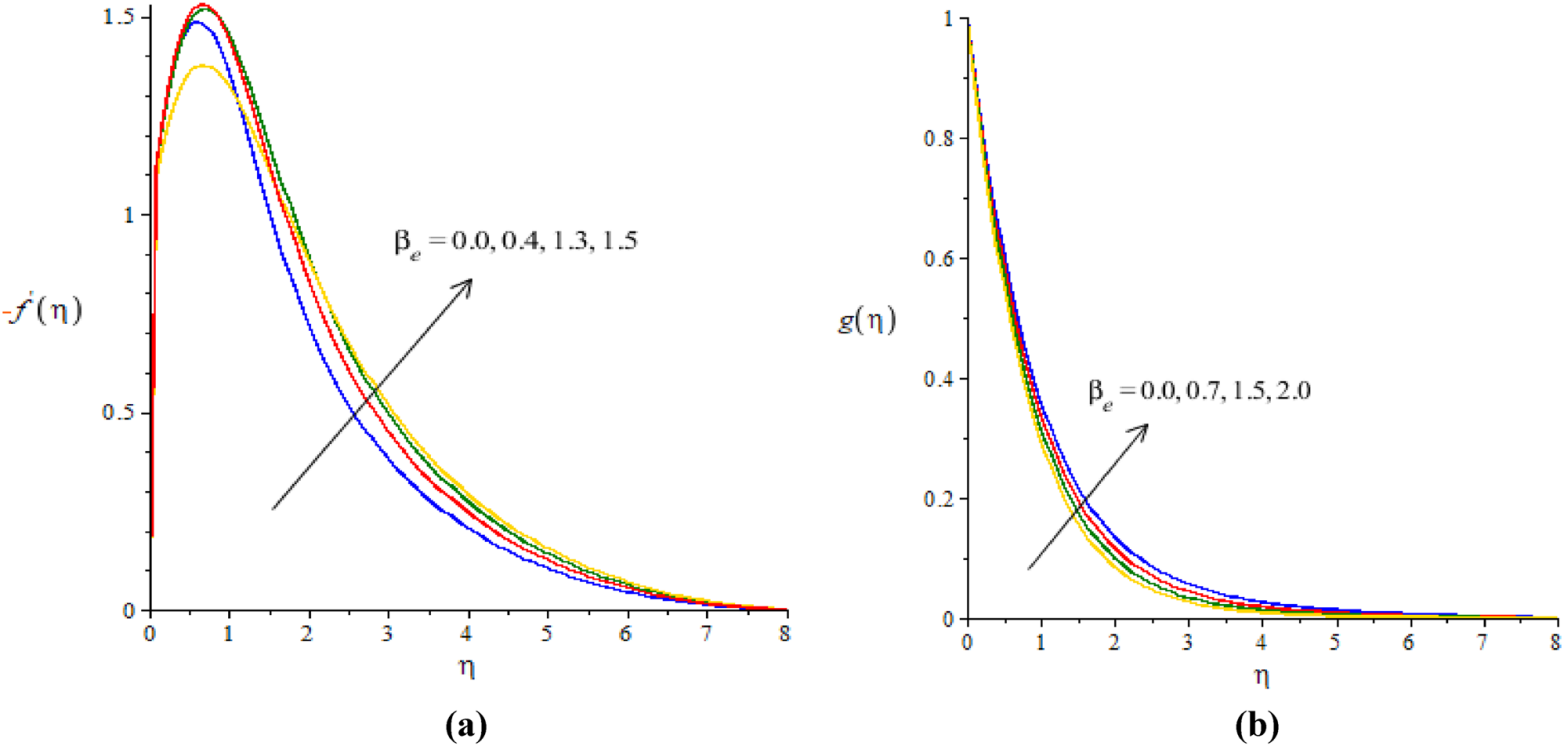
**(a,b)**The graphical view of velocities versus ${\beta }_{e}.$

**Figure 6:**
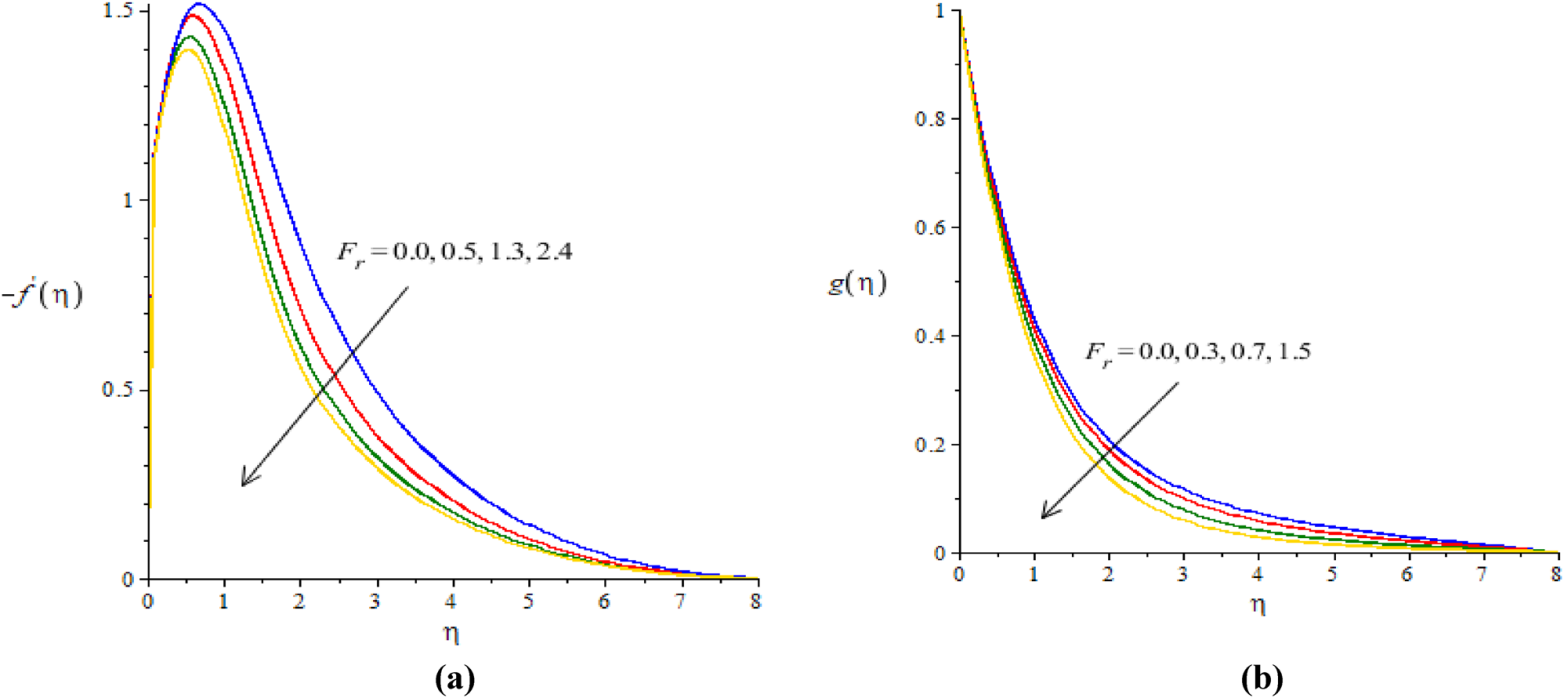
**(a,b)** The graphical view of velocities versus ${F}_{r}.$

The flow situation is verified against the variation of $We$ (Weissenberg number), ion slip and Hall parameters (${\beta }_{i}, {\beta }_{e}$) and Forchheimer number (${F}_{r}$) by Figs. 3a,b, 4a,b, 5a,b, 6a,b. The role of $We$ on the fluid motion is visualized by Fig. 3a,b. The decreasing function is investigated between the relation of $We$ and motion of fluid particles. This decreasing function is plotted using the concept of Weissenberg number carrying the study of nanoparticles and hybrid nanoparticles. Physically, Weissenberg number has direct relation versus elastic force whereas Weissenberg number has inverse relation against viscous force. An increment in Weissenberg number creates more viscosity in fluid particles. More viscous fluid is occurred against large values of Weissenberg number. Therefore, reduction in motion of nanoparticles and hybrid nanoparticles is captured. Figure 4a,b visualize the flow behavior versus the change in ion-slip number. It is noticed that ${\beta }_{i}$ appears in momentum equations reveals the direct relation versus the motion of fluid particles. An increase in ${\beta }_{i}$ results more enhancement in motion of fluid particles is occurred. So, ionization of particles is useful to develop speed in fluid particles. The concept of ion-slip parameter is formulated using generalized ohm’s law. The collision due to ions into fluid particles is enhanced when ion-slip number is inclined. Further, ion-slip number has inverse relation with respect to Lorentz force. So, higher values of ion-slip number create reduction in Lorentz force. Reduction in Lorentz force makes an increment in motion of fluid particles. Hence, ${\beta }_{i}$ is favorable number to obtain the maximum speed in motion of fluid particles. ${\beta }_{e}$ is called Hall parameter and physical situation is taken out on the flow considering by Fig. 5a,b. Same situation is captured for the case of Hall parameter towards the motion in fluid particles. In physical point of view, Hall parameter has significant role on the motion of fluid particles. The fluid motion accelerates versus the impact of ${\beta }_{e}.$ It is noticed that inverse relation is investigated among frictional magnetic force and Hall force. Lorentz force is also reduced versus higher values of Hall force. Such kinds of happenings are made reason for reduction into fluid motion. The distribution of ${F}_{r}$ on the flow in view of vertical and horizontal directions is addressed by Fig. 6a,b. The primary and secondary flows are declined versus the change in ${F}_{r}.$ ${F}_{r}$ is modeled due to concept of Forchheimer porous media into fluid particles. ${F}_{r}$ is known as non-linear function versus the motion into fluid particles. The retardation force is formulated against the impact of ${F}_{r}.$ This reduction is produced due to porous surface inserting the parameter ${F}_{r}.$ Hence, decreasing trend is captured into the motion of fluid particles.

### Graphical simulations of fluid temperature

**Figure 7:**
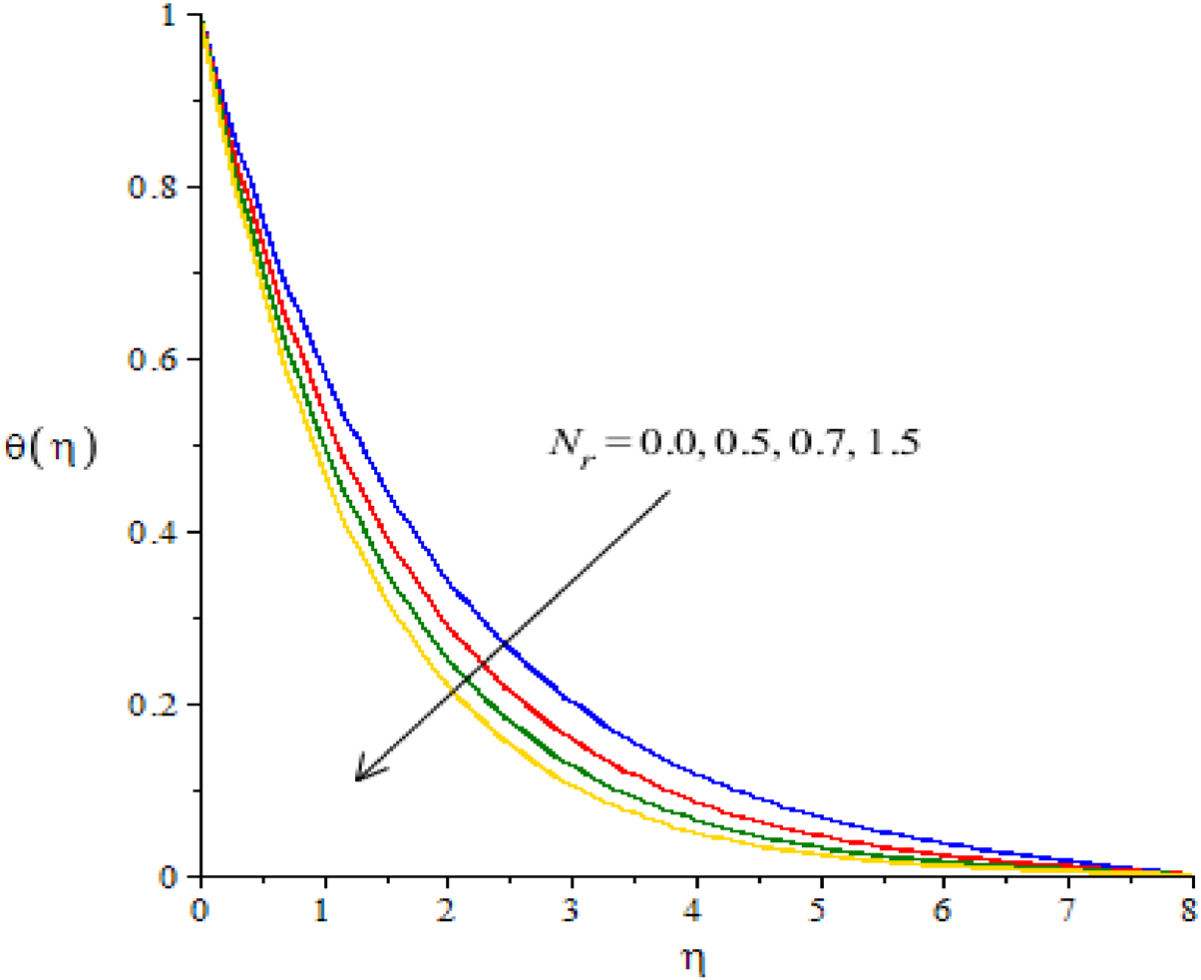
The graphical view of temperature versus ${N}_{r}.$

**Figure 8:**
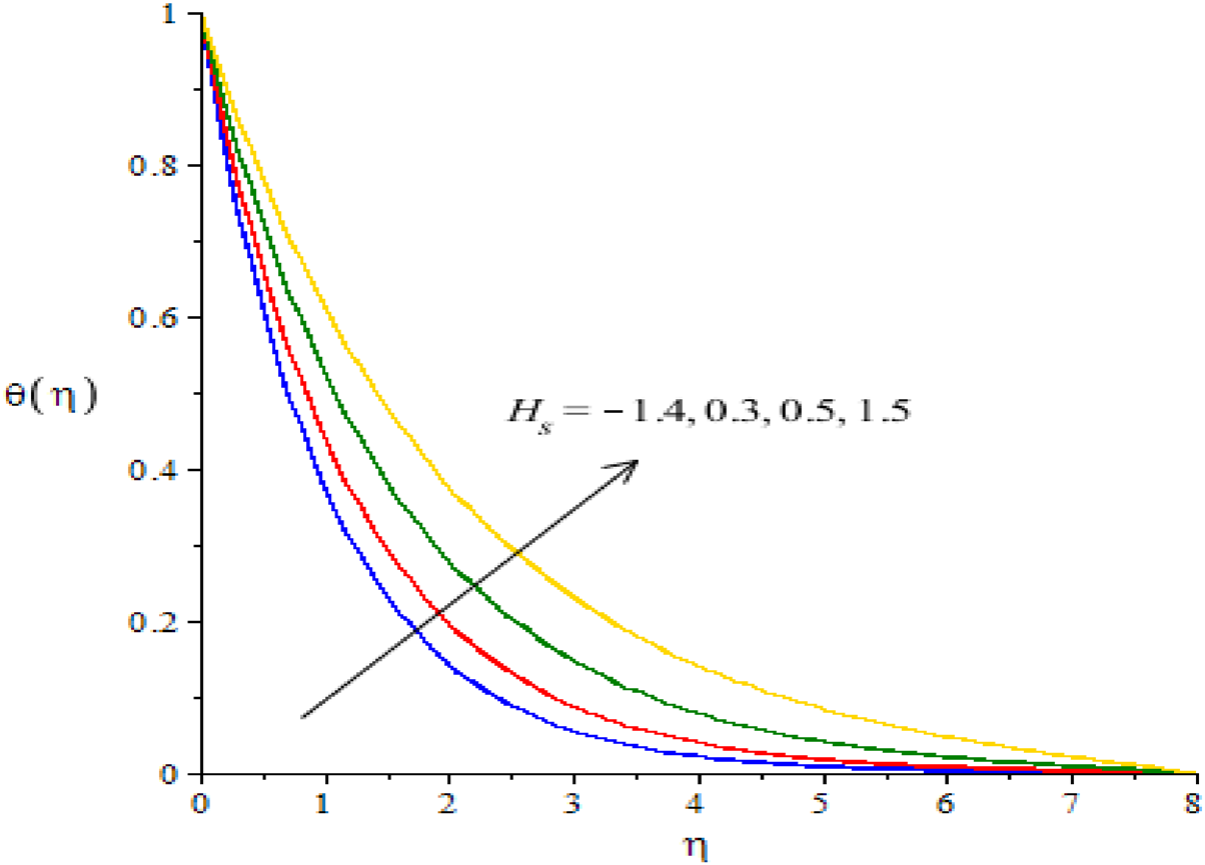
The graphical view of temperature versus ${H}_{s}.$

**Figure 9:**
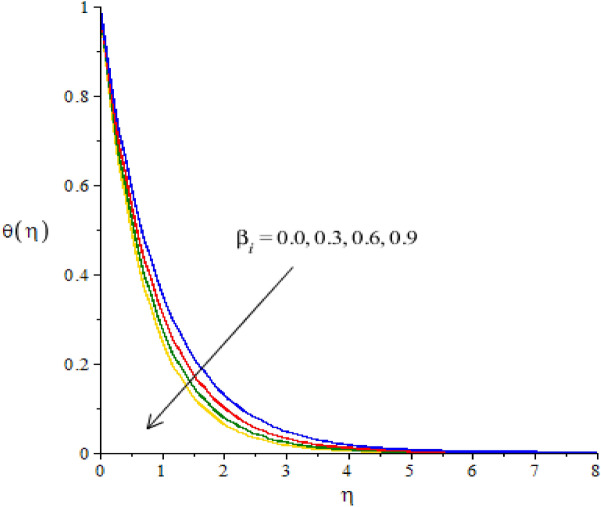
The graphical view of temperature versus ${\beta }_{i}.$

**Figure 10:**
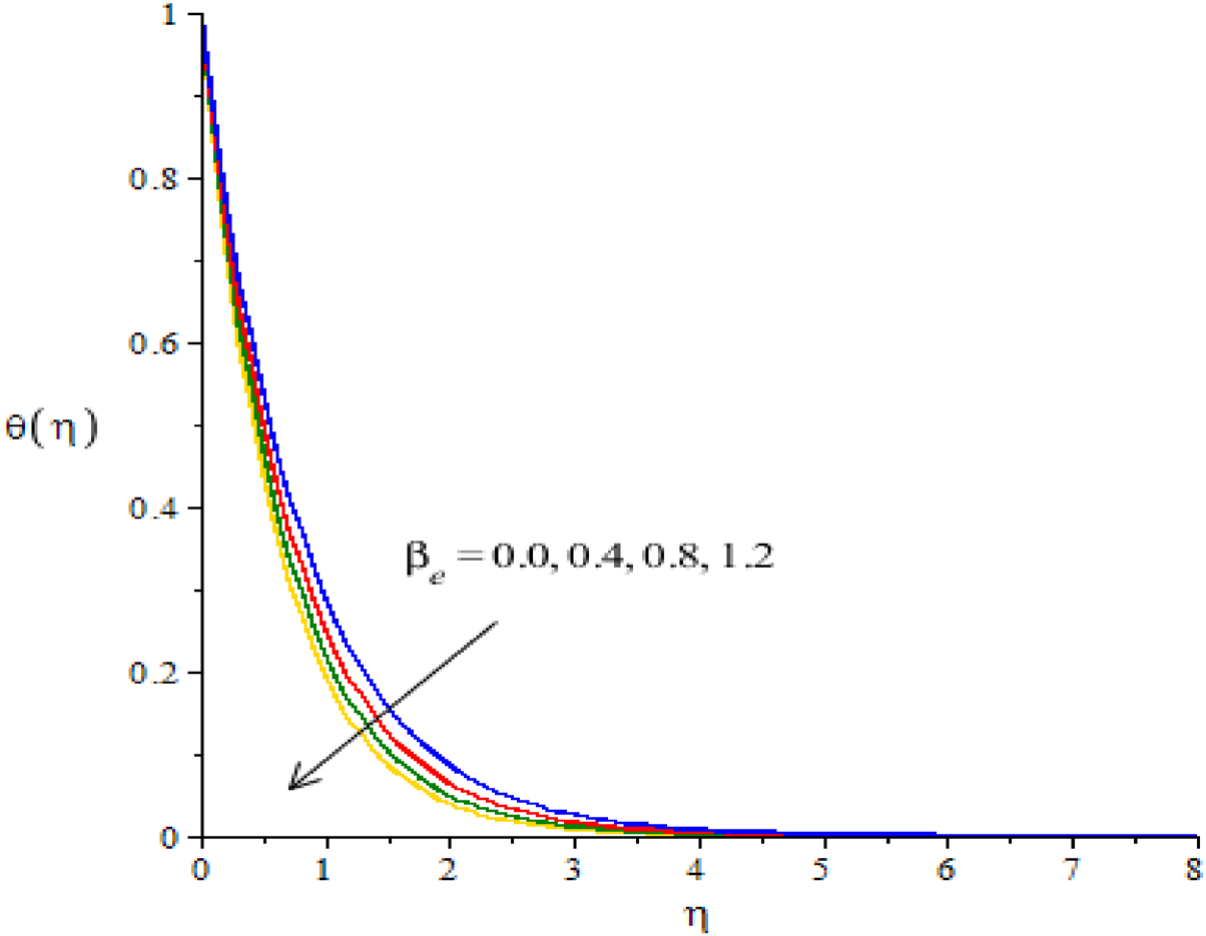
The graphical view of temperature versus ${\beta }_{e}.$

**Figure 11:**
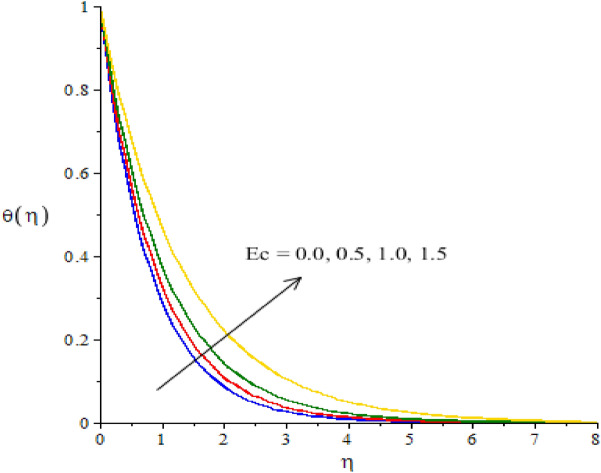
The graphical view of temperature versus $Ec.$

The phenomenon of fluid temperature is addressed against the variation of ${N}_{r}, {H}_{s}, {\beta }_{e}, {\beta }_{i}$ and $Ec.$ The graphical role of fluid temperature inserting nanoparticles and hybrid nanoparticles is measured by Figs. 7, 8, 9, 10 and 11. Figure 7 illustrates the behavior of ${N}_{r}$ on the fluid temperature. In this plotting graph, the reduction is simulated in view of thermal energy. This reduction is created due to large values of thermal radiation number. Physically, heat energy moves away from the surface of cone in form of electromagnetic waves. By this impact, the reduction is occurred into heat energy of fluid particles. Moreover, inverse relation is modeled among thermal radiation and heat energy. Large values of thermal radiation number make reduction in heat energy of hybrid nanoparticles. The character of ${H}_{s}$ versus the temperature profile is considered by Fig. 8. The large values of heat energy make the more production in heat energy while large amount of heat energy is made due to external heat source. Hence, external heat source makes the reason for obtaining the maximum production of heat energy. It is noticed that negative values of ${H}_{s}$ are taken due to heat absorption while positive values for ${H}_{s}$ are indicated as concept of heat generation. More heat energy generates using the concept of external heat source. Figures 9 and 10 reveal the relation between fluid temperature and ion slip and Hall numbers. The production of heat energy is decreased via enlargement in ion slip and Hall numbers. It is estimated that ion slip and Hall numbers are appeared in energy equation. Moreover, the inverse relation is captured versus the existence of ion slip and Hall numbers. An increment in ion slip and Hall numbers brings the reduction in heat energy. It is mentioned that ion slip and Hall currents are formulated due to concept of Joule heating phenomena (in the attendance of generalized ohm’s theory). Joule heating phenomena indicates inverse relation against ion slip and Hall currents. Hence, Joule heating is inclined using higher values of ion slip and Hall currents. MBLT (thickness of momentum boundary layers) are adjusted by varying values of ion slip and Hall currents. The motion of ions makes reduction in heat energy due to large values of ion slip and Hall numbers. The characterization of $Ec$ is considered as an essential role for maximum achievement of thermal energy while this behavior is captured by Fig. 11. From mathematical view, $Ec$ is appeared in energy equation (dimensionless). Hence, direct relation is investigated versus thermal energy. Physically, $Ec$ is modeled due to viscous dissipation in energy equation. More viscous dissipation is developed inserting the role of $Ec$. Heat energy dissipates when viscous nature is occurred into fluid particles. Maximum heat energy is produced because of additional retarding force. Meanwhile, $Ec$ is visualized as a useful parameter for developing more thermal energy.

### Numerical treatment of surface force and Nusselt number

The surface force, temperature gradient and Sherwood number are visualized near the surface of cone. The numerical simulations of surface force, Nusselt number and rate of solute against change in $We, {F}_{r}, {\beta }_{e}, {\beta }_{i}$ and ${H}_{s}$ are simulated. These numerical simulations are taken out by Table 4. The surface force is decreased via enlargement of heat generation, ion slip and Hall numbers but surface force called the skin friction coefficient is significantly enhanced considering variation of ${F}_{r}.$ The production of rate of heat energy is increased versus the enhancement of Forchheimer, ion slip and Hall numbers. Therefore, Forchheimer, ion slip and Hall numbers play a vital impact for the enhancement of temperature gradient. In case of heat generation number gradient temperature is reduced due to inserting the large values of heat generation number.

**Table 4:** Numerical values of gradient temperature and skin friction coefficients versus various parameters.

		${-\left(Re\right)}^{1/2}{C}_{f}$	${-\left(Re\right)}^{1/2}{C}_{g}$	${-\left(Re\right)}^{-1/2}Nu$
$We$	0.0	0.4998714299	0.6029282911	0.7104807445
	0.3	0.8581100631	0.8821689645	0.7114238688
	0.5	2.698548989	0.9686090229	0.7121467218
${F}_{r}$	0.0	0.2864470301	0.7360866944	0.7111316028
	0.7	0.2704802904	0.8166854253	0.7098459854
	1.3	0.2277144057	0.8787076034	0.7080141595
${\beta }_{e}$	0.0	0.2713783769	0.1613967311	0.7119591618
	0.3	0.2680990013	0.13730063316	0.8132986239
	0.7	0.2398120967	0.02406085455	0.9137297415
${\beta }_{i}$	0.0	0.2753085726	0.2499991558	0.7122827047
	0.4	0.1787392657	0.1207572825	0.8099581562
	0.8	0.0824121081	0.0860598593	0.9398457277
	− 1.3	0.2826682046	0.7000830123	0.17668466241
${H}_{s}$	0.0	0.1839467002	0.5539122359	0.36947614874
	1.2	0.00849283892	0.3058238772	0.71907271478

## Key consequences of current model

The porous and rotating cone is used to visualize the impacts of ion slip and Hall forces in thermal energy mechanism considering Carreau Yasuda liquid. The phenomenon of heat transport is occurred in the presence of heat generation, nanoparticles, hybrid nanoparticles and thermal radiation. The numerical scheme (FEM) is used to simulate the numerical results. The prime consequences discussed below.

- Hall and ion slip parameters are considered significant parameters to produce the enhancement in motion of fluid particles but speed of nano and hybrid nanoparticles becomes slow down versus large values of Forchheimer and Weissenberg numbers;
- An enhancement in production of heat energy is addressed via large values of heat generation number and Eckert number while reduction in heat energy is occurred due to positive values of thermal radiation and Hall and ion slip parameters;
- The convergence analysis is simulated via 300 elements;
- The temperature gradient is enhanced against the enhancement in Forchheimer, ion slip and Hall parameters but reverse behavior in noticed for the case of heat generation number;
- Surface force is improved neat wall of cone with respect to variation in Forchheimer number. Surface force is declined via higher values of heat generation and ion slip and Hall parameters;
- Dimensionless stresses and heat transfer coefficient varies directly against Weissenberg parameter.

## Data Availability

The datasets generated/produced during and/or analyzed during the current study/research are available from the corresponding author on reasonable request.
